# Real-World Effectiveness of Rosuvastatin–Ezetimibe Single Pill (Rovazet^®^) in Korean Dyslipidemia Patients

**DOI:** 10.3390/jcm14155480

**Published:** 2025-08-04

**Authors:** Hack-Lyoung Kim, Hyun Sung Joh, Sang-Hyun Kim, Myung-A Kim

**Affiliations:** Division of Cardiology, Department of Internal Medicine, Boramae Medical Center, Seoul National University College of Medicine, 5 Boramae-ro, Dongjak-gu, Seoul 07061, Republic of Korea; wingx4@naver.com (H.S.J.); shkimmd@snu.ac.kr (S.-H.K.); kma@snu.ac.kr (M.-A.K.)

**Keywords:** dyslipidemia, ezetimibe, observational study, rosuvastatin, safety

## Abstract

**Background:** Fixed-dose combinations of rosuvastatin and ezetimibe are increasingly used in clinical practice, but real-world data on their effectiveness and safety in large populations remain limited. **Methods:** This prospective, single-group, open-label, non-interventional observational study was conducted in the Republic of Korea to evaluate the effectiveness and safety of Rovazet^®^ (a fixed-dose combination of rosuvastatin and ezetimibe). Patients were prospectively enrolled from 235 institutions (50 general hospitals and 185 private clinics) as part of routine clinical practice over a five-year period. Lipid profiles and medication compliance questionnaire results were collected at baseline, 12 weeks, and 24 weeks of treatment. **Results:** A total of 5527 patients with dyslipidemia, the majority were men (53.0%), and the mean age was 60.4 years. Rovazet^®^ significantly reduced low-density lipoprotein cholesterol (LDL-C) by 23.5% at 12 weeks (from 117.47 ± 50.65 mg/dL to 81.14 ± 38.20 mg/dL; *p* < 0.0001) and by 27.4% at 24 weeks (from 117.47 ± 50.65 mg/dL to 74.52 ± 33.36 mg/dL; *p* < 0.0001). Total cholesterol was significantly reduced by 17.7% at 12 weeks and by 19.8% at 24 weeks. Rovazet^®^ treatment reduced triglycerides by 4.1% at 12 weeks and by 7.2% at 24 weeks. High-density lipoprotein cholesterol increased by 4.5% at 12 weeks and by 7.9% at 24 weeks following Rovazet^®^ treatment. These changes in lipid profiles were consistent, regardless of cardiovascular risk profiles. By 24 weeks of treatment with Rovazet^®^, 91.8% of patients had reached their target LDL-C goals. Adverse drug reactions were reported in 2.81% of patients, most of which were minor, indicating that Rovazet^®^ was well tolerated. **Conclusions:** Rovazet^®^ was effective in improving lipid profiles and well tolerated in Korean adults with dyslipidemia.

## 1. Introduction

Dyslipidemia is a significant risk factor for cardiovascular diseases, which remain a leading cause of morbidity and mortality worldwide [1]. Low-density lipoprotein cholesterol (LDL-C) has been identified as a primary target for lipid-lowering therapy due to its strong correlation with the development of atherosclerosis and subsequent cardiovascular events [2,3]. Effective management of LDL-C levels is, therefore, crucial in reducing the risk of these conditions [2,3].

Rosuvastatin, a potent statin, and ezetimibe, a cholesterol absorption inhibitor, are two well-established lipid-lowering agents that have been extensively studied and proven effective in managing hyperlipidemia. Statins like rosuvastatin work by inhibiting (3S)-hydroxy-3-methylglutaryl-CoA reductase, a key enzyme in the cholesterol biosynthesis pathway, leading to a significant reduction in LDL-C levels [4]. On the other hand, ezetimibe lowers cholesterol levels by inhibiting its absorption in the small intestine, providing a complementary mechanism to that of statins [5]. The combination of these two drugs in a single pill has been developed to enhance patient compliance, reduce the pill burden, and improve therapeutic outcomes by targeting different pathways in cholesterol metabolism [6,7,8,9]. This combination therapy not only simplifies the medication regimen but also maximizes the LDL-C-lowering effect, which is crucial for patients who need intensive lipid management. 

Previous clinical trials have demonstrated the efficacy of rosuvastatin and ezetimibe in lowering LDL-C levels, both as monotherapies and in combination [6,7,8,9,10,11]. However, real-world data on the effectiveness and safety of a fixed-dose combination of rosuvastatin and ezetimibe in a large population is limited [12]. This study aims to fill this gap by providing comprehensive real-world evidence on the LDL-C-lowering effects and safety profile of the drug in a substantial cohort of patients with dyslipidemia.

## 2. Materials and Methods

### 2.1. Study Protocol and Population

**Figure 1:**
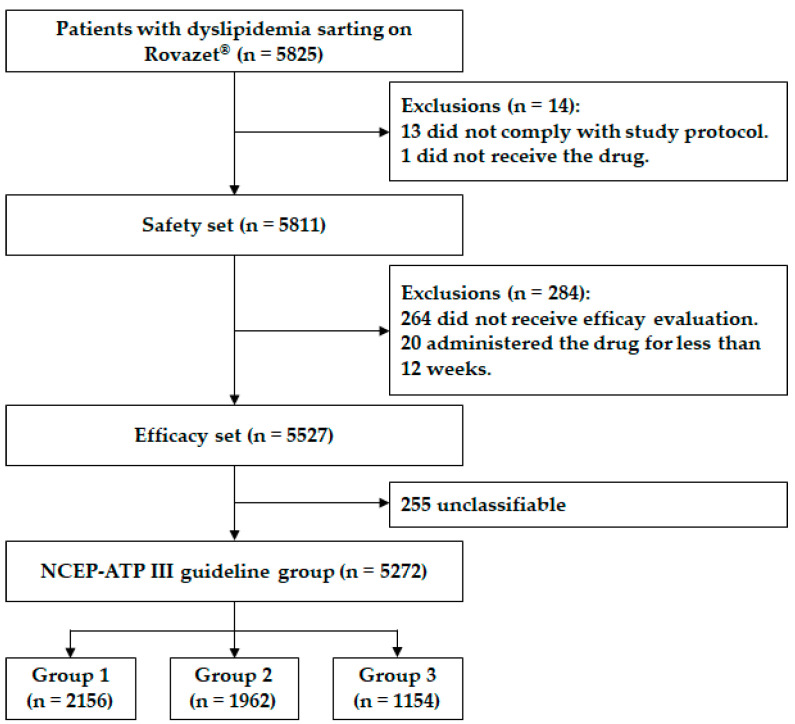
Flow chart for study enrollment. NCEP-ATP, National Cholesterol Education Program Adult Treatment Panel.

Patients were prospectively enrolled at 235 institutions (50 general hospitals and 185 private clinics) in the Republic of Korea over a period of five years, from January 2017 to December 2021. Patients who started using Rovazet^®^ (a fixed-dose combination of rosuvastatin and ezetimibe, HK inno.N Corp., Gyeonggi-do, Republic of Korea) for dyslipidemia were enrolled in the study after providing informed consent. The criteria for study enrollment included Korean adult men and women aged 19 years or older who had been confirmed to be receiving Rovazet^®^ in a routine clinical setting. The dosage of Rovazet^®^ was 10 mg for ezetimibe and 5, 10, or 20 mg for rosuvastatin. The specific dosage of rosuvastatin was determined by the physician based on the patient’s LDL-C level and risk profile. On the day of starting Rovazet^®^, a blood test was performed before taking the drug, and lipid profile data and a medication compliance questionnaire were collected. These lipid profile data and medication compliance questionnaire results were also obtained at 12 and 24 weeks of medication use. Initially, a total of 5825 patients were screened, and 14 patients were excluded due to non-compliance with the study protocol (*n* = 13) and not receiving the medication (*n* = 1). The remaining 5811 patients were evaluated for the safety of the drug (safety set). After excluding 284 patients who did not receive efficacy evaluation (*n* = 264) or had a short drug administration period of less than 12 weeks (*n* = 20), a total of 5527 patients were included in the efficacy evaluation (efficacy set). Of these, 5272 patients could be grouped into three categories according to the National Cholesterol Education Program Adult Treatment Panel Ⅲ (NCEP-ATP III) guidelines [13], while 255 patients were unclassifiable. Group 1 corresponds to the low-risk category, Group 2 to the moderate-risk category, and Group 3 to the high-risk category for cardiovascular disease risk assessment (Supplementary Table S1). The flow chart for study enrollment is shown in Figure 1. The study adhered to the principles of the Declaration of Helsinki and was approved by the Institutional Review Board (IRB) of Boramae Medical Center (Seoul, Republic of Korea) (IRB number: 10-2017-41). Signed consent forms were obtained from all participants before the study commenced.

### 2.2. Clinical Data Collection

Body mass index (BMI) was calculated as body weight (kg) divided by height (m^2^). Systolic and diastolic blood pressures were measured using an automatic oscillometric device. Hypertension was defined based on a previous diagnosis, systolic blood pressure ≥ 140 mmHg, diastolic blood pressure ≥ 90 mmHg, or the use of anti-hypertensive medications. Diabetes mellitus was defined based on a previous diagnosis, fasting glucose ≥ 126 mg/dL, glycated hemoglobin ≥ 6.5%, or the use of anti-diabetic medications. Coronary heart disease included angina pectoris, myocardial infarction, significant stenosis (≥50%), or the presence of myocardial ischemia on imaging studies. Heart failure and stroke were defined based on previous diagnoses by a physician. Family history of cardiovascular disease (coronary heart disease, carotid artery disease, peripheral arterial occlusive disease, and abdominal aortic aneurysm) and diabetes mellitus was obtained. Patients who smoked regularly at the time of study enrollment were considered current smokers. Information on prior anti-dyslipidemic medications, including statins, fibric acid, omega-3 fatty acids, and ezetimibe, was also obtained.

### 2.3. Lipid Profile

The blood lipid profile was checked immediately before taking Rovazet^®^ and again at 12 and 24 weeks after starting the medication. The patient fasted for more than 12 h before venous blood was drawn to measure the levels of total cholesterol (TC), LDL-C, high-density lipoprotein cholesterol (HDL-C), and triglycerides (TG).

### 2.4. Grouping According to the NCEP-ATP III Guidelines

Patients were divided into three groups according to the NCEP-ATP III guidelines, which were calculated based on their cardiovascular risk profiles. The efficacy of Rovazet^®^ was then analyzed across these groups. The specific NCEP-ATP III criteria used for grouping are provided in Supplementary Table S1 [13].

### 2.5. LDL-C Target

The LDL-C target was also based on the LDL-C target for each group presented in the NCEP-ATP III guideline (Supplementary Table S1).

### 2.6. Statistical Analysis

In this study, clinical information was obtained over a period of 5 years from hospitals and clinics in the Republic of Korea. The target was 6092 patients to result in 5483 assessable patients for the study. It was calculated by assuming a 99% confidence level (*α* = 0.01) and applying an expected proportion of 0.91, managing the blood lipid profiles by taking the Rovazet^®^ according previous study [14]. Baseline and clinical characteristics were summarized as means and standard deviations for continuous variables, and numbers and percentages for categorical variables. The significance of percentage changes in LDL-C and other lipid profiles after 12 and 24 weeks was evaluated using a paired *t*-test or Wilcoxon’s signed rank test. The percentage change in LDL-C and other lipid profiles between the groups was evaluated using analysis of variance or Kruskal–Wallis test. McNemar’s test was performed to assess whether there were differences in the percentage of patients reaching the prespecified goals of LDL-C levels according to the NCEP-ATP III guideline between 12 and 24 weeks. The adverse event was summarized as numbers, percentages, and 95% confidence intervals. For all analyses, a *p*-value < 0.05 was considered statistically significant. All statistical analyses were conducted using SAS version 9.4 (SAS Institute Inc., Cary, NC, USA).

## 3. Results

Baseline characteristics of the study patients are shown in Table 1. The mean age was 60.4 ± 11.6 years, and 53.0% were male. The mean BMI was 25.4 ± 3.6 kg/m^2^, indicating that the study patients were mildly obese. Systolic and diastolic blood pressure were within the normal range. The prevalence of hypertension, diabetes mellitus, coronary heart disease, heart failure, and ischemic stroke was 51.2%, 30.0%, 20.1%, 4.6%, and 5.0%, respectively. Additionally, 13.2% of patients had a family history of cardiovascular disease or diabetes mellitus, and 19.5% were current smokers. Approximately half (55.7%) of the patients were taking statins before starting Rovazet^®^.

**Figure 2:**
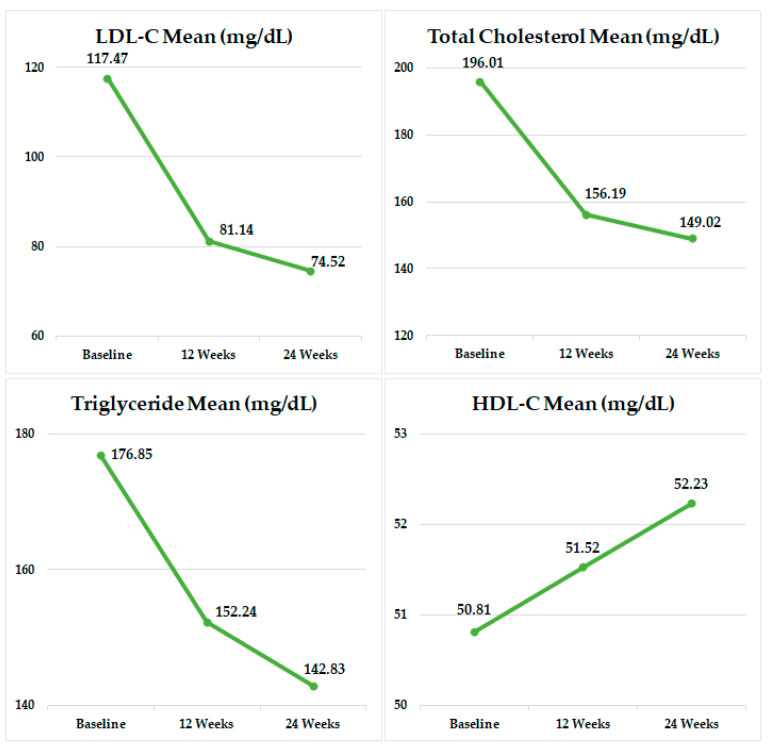
Changes in mean lipid profile values after 12 and 24 weeks of treatment with Rovazet^®^. LDL, low-density lipoprotein; HDL, high-density lipoprotein.

**Figure 3:**
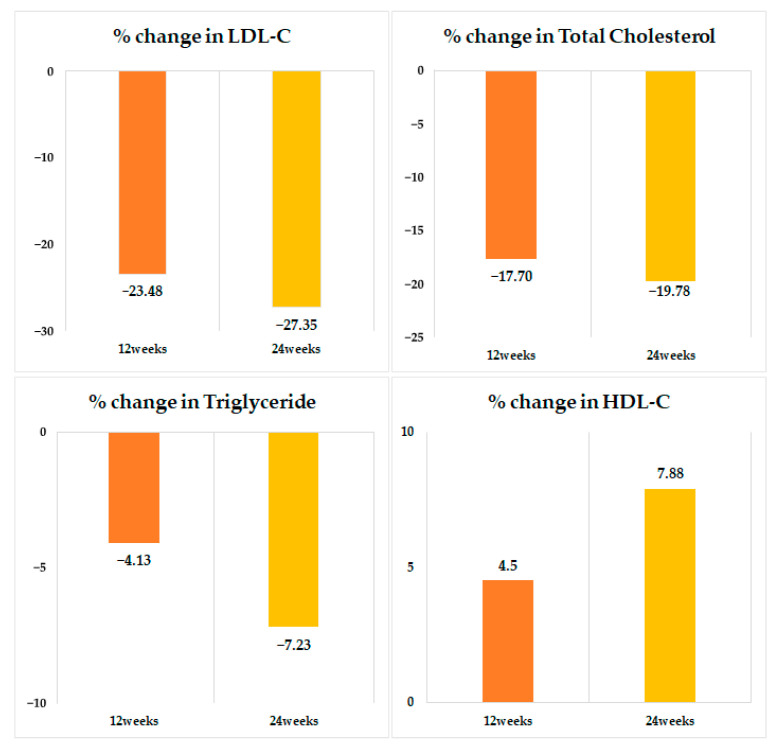
Percent changes in lipid profiles after 12 and 24 weeks of treatment with Rovazet^®^. LDL, low-density lipoprotein; HDL, high-density lipoprotein.

**Figure 4:**
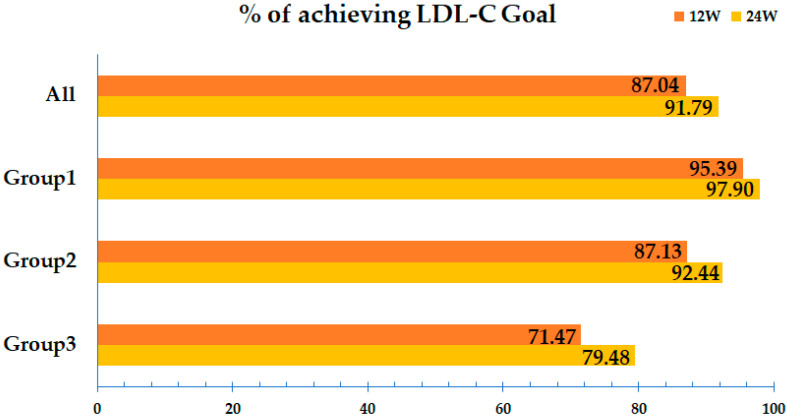
Percent of achieving LDL-C goal. LDL, low-density lipoprotein; HDL, high-density lipoprotein.

Table 2 shows the changes in lipid profiles after 12 and 24 weeks of treatment with Rovazet^®^. In total study population, treatment with Rovazet^®^ significantly reduced LDL-C by 23.4% at 12 weeks (from 117 ± 51 mg/dL to 81.1 ± 38.2 mg/dL; *p* < 0.0001) and by 27.3% at 24 weeks (from 117 ± 51 mg/dL to 74.5 ± 33.3 mg/dL; *p* < 0.0001). TC was significantly reduced by 17.7% at 12 weeks (from 196 ± 59 mg/dL to 156 ± 46 mg/dL; *p* < 0.0001) and by 19.7% at 24 weeks (from 196 ± 59 mg/dL to 149 ± 39 mg/dL; *p* < 0.0001). Rovazet^®^ treatment reduced TG by 4.1% at 12 weeks (from 176 ± 110 mg/dL to 152 ± 100 mg/dL; *p* < 0.0001) and by 7.2% at 24 weeks (from 176 ± 110 mg/dL to 142 ± 82 mg/dL; *p* < 0.0001). HDL-C increased by 4.5% at 12 weeks (from 50.8 ± 15.5 mg/dL to 51.5 ± 15.5 mg/dL; *p* < 0.0001) and by 7.8% at 24 weeks (from 50.8 ± 15.5 mg/dL to 52.2 ± 15.9 mg/dL; *p* < 0.0001) following Rovazet^®^ treatment. At both the 12 and 24 weeks, patients with no history of prior anti-dyslipidemic medication *(n* = 3511) demonstrated greater reductions in LDL-C, TC, and TG levels compared to those with a history of prior anti-dyslipidemic medication group (*n* = 2015), and the change rates for LDL-C, TC, and TG between the two groups were statistically significant (*p* < 0.0001 for each). Furthermore, HDL-C levels showed statistically significant increases in both groups at 12 and 24 weeks; however, the difference between the two groups was not statistically significant (*p* > 0.05) (Supplementary Table S2). Figure 2 and Figure 3 schematically illustrate the changes in lipid profiles at 12 and 24 weeks of Rovazet^®^ use in the total population. These changes in lipid profiles were consistent across all three groups. The LDL-C target attainment rates for each group are shown in Figure 4. At 24 weeks, the LDL-C attainment rates were 97.9% in Group 1, 92.4% in Group 2, and 79.5% in Group 3, indicating that as cardiovascular risk increased, the LDL-C attainment rates were relatively lower.

Table 3 presents the adverse events and adverse drug reactions observed during the study period. In the safety set analysis (*n* = 5811), a total of 588 adverse events in 419 patients (7.21%) and 193 adverse drug reactions in 163 patients (2.81%) were reported. Among these, serious adverse events occurred in 35 patients (0.60%), and serious adverse drug reactions occurred in 4 patients (0.07%). The top 10 adverse events and adverse drug reactions are detailed in Table 4. The most common adverse event was dizziness, occurring in 31 patients (0.53%). Myalgia and abnormal hepatic function were observed in 27 (0.46%) and 23 patients (0.40%), respectively. The most common adverse drug reaction was hypertriglyceridemia, which occurred in 27 patients (0.46%). Abnormal hepatic function and myalgia each occurred in 20 patients (0.34%).

## 4. Discussion

The results of this study demonstrate that Rovazet^®^, a fixed-dose combination of rosuvastatin and ezetimibe, is highly effective in improving lipid profiles among patients with a range of cardiovascular risk factors. The significant reductions in LDL-C, TC, and TG observed at both 12 and 24 weeks highlight Rovazet^®^’s potential as a potent lipid-lowering agent. The increase in HDL-C further suggests its role in enhancing protective lipid factors. These improvements were consistent across all patient groups, indicating that Rovazet^®^ could be beneficial for a wide spectrum of individuals, from those with mild obesity and controlled blood pressure to those with significant comorbidities such as hypertension, diabetes, and coronary heart disease. In terms of safety, the study showed that Rovazet^®^ is generally well tolerated. The incidence of adverse events and adverse drug reactions was relatively low, with serious adverse events being rare.

Recent developments in the use of fixed-dose combinations of statins and ezetimibe have shown promising results in managing cardiovascular risk by effectively lowering LDL-C. Studies have demonstrated that these combinations can provide greater reductions in LDL-C levels compared to statin monotherapy [6,8,9], which is crucial for patients who require more aggressive lipid-lowering therapy to meet their cholesterol goals. This enhanced efficacy is likely due to the complementary mechanisms of action: while statins inhibit cholesterol synthesis in the liver, ezetimibe blocks cholesterol absorption in the intestines [5]. This dual approach not only lowers LDL-C but also reduces the risk of cardiovascular events, such as myocardial infarction and stroke [15,16]. Furthermore, combination therapy offers improved patient adherence due to the convenience of a single pill [12], which may contribute to better long-term outcomes. The safety profiles for these fixed-dose combinations are generally comparable to those of monotherapy, with most studies reporting low rates of adverse effects, making them a viable option for long-term management of dyslipidemia [7,15]. Although our study was a single-arm observational study and cannot be directly compared with randomized controlled trials like those involving rosuvastatin, the real-world evidence of the efficacy and safety of Rovazet^®^ that we have demonstrated is noteworthy. We also confirmed the effectiveness of Rovazet^®^ in patients with different cardiovascular risk profiles by enrolling a large number of patients. These findings underscore the potential of Rovazet^®^ as an effective treatment option for lipid management in diverse patient populations.

Dividing patients into three groups based on the NCEP-ATP III criteria provides a scientifically justified and clinically relevant approach for assessing the effectiveness of cholesterol-lowering therapy. The NCEP-ATP III guidelines are widely recognized and validated for managing dyslipidemia and stratifying cardiovascular risk [13]. These guidelines categorize patients into different risk groups based on their lipid profiles, the presence of cardiovascular risk factors, and their overall risk of developing coronary heart disease. By applying the NCEP-ATP III criteria to create three distinct groups, we ensure that each group represents a different level of cardiovascular risk. This stratification allows for a more tailored evaluation of the effectiveness of statin therapy, as the response to statins can vary significantly depending on the baseline risk and lipid levels of the patient. Furthermore, it enables a more nuanced understanding of how statin therapy impacts different patient populations, thereby guiding more personalized treatment strategies. In our study, the LDL-C-lowering effect was similar across all groups. However, the rate of achieving target LDL-C levels was slightly lower in Group 3, the high-risk group, compared to the other groups. This finding suggests that more potent LDL-C-lowering therapies may be necessary for high-risk groups to achieve optimal outcomes.

In terms of safety, the study showed that Rovazet^®^ is generally well tolerated among the patient population. The incidence of adverse events and adverse drug reactions was relatively low, indicating a favorable safety profile. Serious adverse events were rare, further underscoring the drug’s safety. The most commonly reported adverse events were dizziness and myalgia, both of which were mild and manageable, suggesting that these side effects would not significantly hinder patients’ daily activities or adherence to the medication. Additionally, while hypertriglyceridemia was identified as one of the common adverse drug reactions, its incidence was relatively low. This finding suggests that hypertriglyceridemia does not pose a substantial barrier to the widespread use of Rovazet^®^. This supports the feasibility of using Rovazet^®^ for long-term management in clinical practice, as the benefits outweigh the risks associated with these mild adverse effects.

The LDL-C reduction observed in this study (−23.5% at 12 weeks and −27.4% at 24 weeks) is in line with the overall lipid-lowering effect of rosuvastatin/ezetimibe combination therapy reported in previous randomized controlled trials. In the Ildong Rosuvastatin and Ezetimibe for Hypercholesterolemia (I-ROSETTE) study, a multicenter Phase III trial conducted in the Republic of Korea, the combination of rosuvastatin and ezetimibe resulted in a 57.0% reduction in LDL-C, significantly greater than the 44.4% reduction achieved by rosuvastatin monotherapy after 8 weeks (*p* < 0.001) [10]. Similarly, a 24-week randomized trial by Kim et al. demonstrated that rosuvastatin/ezetimibe 10/10 mg provided superior LDL-C reduction compared to high-dose rosuvastatin 20 mg in patients with hypercholesterolemia [17]. Although the magnitude of LDL-C reduction in our observational study was more modest than that reported in RCTs, the results confirm the real-world effectiveness of this combination therapy in a broader and more heterogeneous patient population.

This study demonstrated that co-administration of rosuvastatin and ezetimibe (Rovazet^®^) led to a significant reduction in LDL-C levels by 23.5% at 12 weeks and 27.4% at 24 weeks. While the absolute reduction continued through 24 weeks, the additional decrease between weeks 12 and 24 was relatively modest and may not have reached statistical significance. This trend is consistent with previous studies, in which the majority of LDL-C reduction with combination therapy occurred within the initial 12 weeks. For example, in the study by Obońska et al., a 37–40% reduction in LDL-C was observed at week 12 with rosuvastatin/ezetimibe combination therapy; however, the additional reduction from week 6 to 12 was not statistically significant (*p* = 0.077), suggesting a plateau effect after the early treatment phase [18]. Similarly, a Korean randomized controlled trial comparing rosuvastatin/ezetimibe (10/10 mg) with high-dose rosuvastatin (20 mg) showed a 22.9% LDL-C reduction at 12 weeks and 24.2% at 24 weeks in the combination group, with minimal further decline during the latter period [17]. These findings indicate that the primary lipid-lowering effect of combination therapy is achieved within the first few months, after which LDL-C levels tend to stabilize. Therefore, the lack of a significant change between 12 and 24 weeks in our study should not be interpreted as a failure of therapy, but rather as a well-recognized pharmacodynamic feature of statin–ezetimibe combination treatment. This underscores the rapid onset and sustained efficacy of this therapeutic approach.

While it is true that previous real-world studies have assessed the combination of rosuvastatin and ezetimibe [12,19,20,21], we believe our study contributes meaningfully to the existing body of evidence in several ways. First, most previous studies were retrospective in nature [12,19,20,21], and some were based on analyses of claim data [12,19,21]. In contrast, our study was a prospective observational study that enrolled more than 5500 patients, providing robust data on the effectiveness and safety of the fixed-dose combination in routine clinical practice. Second, our study focuses specifically on a Korean population, which is underrepresented in prior studies [12,19,20,21]. Given known ethnic differences in lipid metabolism and statin response, region-specific data are clinically valuable. Third, we evaluated not only lipid-lowering effects but also medication adherence using a standardized questionnaire, which provides insights into real-world compliance patterns associated with fixed-dose therapy. Lastly, we performed subgroup analyses across different cardiovascular risk profiles, which demonstrated consistent efficacy regardless of baseline risk. Taken together, we believe our study offers clinically relevant and population-specific findings that support the use of fixed-dose rosuvastatin–ezetimibe therapy in everyday practice.

There are several study limitations. The single-arm design of the study limits the ability to make direct comparisons with other treatments or a placebo, introducing potential biases. The relatively short follow-up period may not capture long-term outcomes, including rare adverse events or the sustainability of the lipid-lowering effects. Another limitation of this study is that pharmacogenetic markers known to influence rosuvastatin metabolism and response, such as SLCO1B1 or ABCG2 variants, were not assessed. Given the potential impact of these genetic factors on drug efficacy and safety, future studies incorporating pharmacogenetic data are warranted to provide a more individualized approach to statin therapy. Additionally, the study population may not be representative of all patients who might use Rovazet^®^, which limits the generalizability of the findings. The reliance on observational data may also introduce reporting biases and inaccuracies, affecting the reliability of the results. Potential confounders, such as lifestyle changes or the use of other medications, could have influenced the outcomes, but this data was not available in our study. These limitations suggest the need for further research, including randomized controlled trials with larger sample sizes and longer follow-up, to confirm these findings and provide a more comprehensive understanding of Rovazet^®^’s safety and efficacy in diverse patient populations.

In conclusion, this study demonstrates that Rovazet^®^ is an effective option for improving lipid profiles in patients with a variety of cardiovascular risk factors. Significant reductions in LDL-C, total cholesterol, and triglycerides, as well as increases in HDL-C, were observed over 12 and 24 weeks of treatment. Also, Rovazet^®^ was well tolerated. Future research, particularly randomized controlled trials, is needed to confirm these results.

## Figures and Tables

**Table 1:** Baseline clinical characteristics of study patients.

Characteristic	Value (*n* = 5527)
Age, years	60.4 ± 11.6
Female sex	2599 (47.0)
Body weight, kg	67.9 ± 13.1
Height, cm	163 ± 9
Body mass index, kg/m^2^	25.4 ± 3.6
Systolic blood pressure, mmHg	128 ± 14
Diastolic blood pressure, mmHg	77 ± 10
*Medical history*	
Hypertension	2828 (51.2)
Diabetes mellitus	1660 (30.0)
Coronary heart disease	903 (20.1)
Heart failure	254 (4.6)
Ischemic stroke	293 (5.0)
Family history of cardiovascular disease or diabetes mellitus	731 (13.2)
Current cigarette smoking	1079 (19.5)
*Prior anti-dyslipidemic medications*	
Statin	3246 (55.7)
Fibric acid	70 (1.2)
Omega-3 fatty acid	61 (1.0)
Ezetimibe	7 (0.1)

Numbers are expressed as mean ± standard deviation or *n* (%).

**Table 2:** Changes of cholesterol profiles after 12 and 24 weeks of treatment with Rovazet^®^.

Cholesterol Profile	Efficacy Set (*n* = 5527)						
	Baseline (*n* = 5515)	At 12 Weeks (*n* = 4870)	% Change (*n* = 4861)	*p*	At 24 Weeks (*n* = 4941)	% Change (*n* = 4936)	*p*
*TC,* mg/dL				0.0027 *			0.0001 *
Total population	196 ± 59	156 ± 46	−17.7 ± 21.9	<0.0001	149 ± 39	−19.7 ± 23.2	<0.0001
Group 1 (*n* = 2149)	205 ± 58	161 ± 43	−18.3 ± 21.9	<0.0001	154 ± 37	−20.6 ± 23.0	<0.0001
Group 2 (*n* = 1962)	184 ± 56	149 ± 44	−16.3 ± 21.5	<0.0001	143 ± 38	−18.0 ± 22.5	<0.0001
Group 3 (*n* = 1148)	199 ± 62	157 ± 50	−17.5 ± 22.5	<0.0001	148 ± 44	−20.5 ± 24.6	<0.0001
*LDL-C*, mg/dL				0.0037 *			0.0112 *
Total population	117 ± 51	81.1 ± 38.2	−23.4 ± 87.8	<0.0001	74.5 ± 33.3	−27.3 ± 56.4	<0.0001
Group 1 (*n* = 2154)	123 ± 52	82.9 ± 36.6	−26.5 ± 34.7	<0.0001	76.3 ± 31.7	−28.9 ± 36.4	<0.0001
Group 2 (*n* = 1959)	110 ± 48	78.8 ± 39.1	−19.2 ± 140	<0.0001	72.6 ± 33.5	−24.4 ± 79.5	<0.0001
Group 3 (*n* = 1153)	116 ± 50	81.1 ± 39.3	−24.1 ± 36.6	<0.0001	73.9 ± 35.9	−28.2 ± 41.1	<0.0001
*TG*, mg/dL				<0.0001 *			<0.0001 *
Total population	176 ± 110	152 ± 100	−13.0 ± −92.0	<0.0001	142 ± 82	−7.2 ± 53.7	<0.0001
Group 1 (*n* = 2156)	153 ± 86	138 ± 75	−0.81 ± 53.4	<0.0001	132 ± 77	−3.22 ± 52.0	<0.0001
Group 2 (*n* = 1962)	190 ± 119	162 ± 125	−4.79 ± 59.3	<0.0001	150 ± 84	−8.58 ± 58.7	<0.0001
Group 3 (*n* = 1153)	192 ± 127	159 ± 95	−7.34 ± 44.8	<0.0001	149 ± 84	−10.1 ± 49.3	<0.0001
*HDL-C*, mg/dL				<0.0001 *			<0.0001 *
Total population	50.8 ± 15.5	51.5 ± 15.5	4.50 ± 29.9	<0.0001	52.2 ± 15.9	7.88 ± 50.0	<0.0001
Group 1 (*n* = 2156)	58.5 ± 16.0	56.7 ± 13.7	−0.59 ± 20.9	<0.0001	57.0 ± 14.2	0.99 ± 45.9	0.0158
Group 2 (*n* = 1962)	45.4 ± 12.7	48.1 ± 17.3	8.31 ± 36.9	<0.0001	49.1 ± 18.1	13.3 ± 61.2	<0.0001
Group 3 (*n* = 1150)	47.1 ± 13.6	48.7 ± 13.5	6.39 ± 30.0	<0.0001	49.5 ± 13.2	9.17 ± 36.1	<0.0001

* Paired *t*-test or Wilcoxon’s signed rank test (within group), ANOVA or Kruskal–Wallis test (between group). Numbers are expressed as mean ± standard deviation. TC, cholesterol; LDL-C, low-density lipoprotein cholesterol; TG, triglyceride; HDL-C, high-density lipoprotein cholesterol.

**Table 3:** Adverse events and adverse drug reactions.

	Safety Set (*n* = 5811)		
	*n* (%)	95% CI	Frequency
Adverse event	419 (7.21)	6.56–7.91	558
Adverse drug reaction	163 (2.81)	2.40–3.26	193
Serious adverse event	35 (0.60)	0.42–0.84	38
Serious adverse drug reaction	4 (0.07)	0.02–0.18	4

**Table 4:** The number of top 10 adverse events and adverse drug reactions based on World Health Organization Adverse Reaction Terminology.

Terminology	Safety Set (*n* = 5811)	
	*n* (%)	Frequency
*Adverse event*		
Dizziness	31 (0.53)	31
Hypertriglyceridemia	28 (0.48)	28
Myalgia	27 (0.46)	29
Abnormal hepatic function	23 (0.40)	23
Chest pain	21 (0.36)	21
Headache	20 (0.34)	21
Abdominal pain	14 (0.24)	14
Dyspepsia	14 (0.24)	14
Conspitation	12 (0.21)	12
Gastroesophageal reflux disease	12 (0.21)	12
*Total*	202 (3.48)	205
*Adverse drug reaction*		
Hypertriglyceridemia	27 (0.46)	27
Abnormal hepatic function	20 (0.34)	20
Myalgia	20 (0.34)	20
Dizziness	10 (0.17)	10
Hyperlipidemia	7 (0.12)	7
Hypercholesterolemia	7 (0.12)	7
Gastroesophageal reflux disease	6 (0.10)	6
Headache	4 (0.07)	4
Chest pain	4 (0.07)	4
Insomnia	4 (0.07)	4
*Total*	109 (1.88)	109

## Data Availability

The datasets generated and analyzed during the current study are available from the corresponding author on reasonable request.

## Supplementary Materials

The following supporting information can be downloaded at: https://www.mdpi.com/article/10.3390/jcm14155480/s1, Table S1: Grouping and LDL-C targets in NCEP-ATP Ⅲ guideline; Table S2: Changes in cholesterol profiles after 12 and 24 weeks of treatment with Rovazet® according to prior use of anti-dyslipidemic medication versus no prior use (treatment-naïve).
