# Supplementary material for: Real-World Effectiveness of Rosuvastatin–Ezetimibe Single Pill (Rovazet®) in Korean Dyslipidemia Patients
Source: J Clin Med. 2025 Aug 4;14(15):5480. doi: 10.3390/jcm14155480 (PMC12347665; doi:10.3390/jcm14155480)
Supplement: Supplementary file 1 [file jcm-14-05480-s001.zip › jcm-3761382-supplementary.pdf]

Supplementary Table S1. Grouping and LDL-C targets in NCEP-ATP Ⅲ guideline.

Risk category	Group	LDL-C target (mg/dL)
0–1 risk Factor	Group 1	< 160
2 + risk Factor (10-year risk ≤ 20%)<br>Multiple risk factors that confer a 10-year risk for CHD ≤ 20%.<br>(Risk is estimated from Framingham risk scores.)	Group 2	< 130
CHD or CHD risk equivalents (10-year risk > 20%) *<br>Other clinical forms of atherosclerotic disease (peripheral arterial disease, abdominal aortic aneurysm, and symptomatic carotid artery disease)<br>Diabetes mellitus<br>Multiple risk factors that confer a 10-year risk for CHD > 20%.<br>(Risk is estimated from Framingham risk scores.)	Group 3	< 100
Major Risk Factors		
Cigarette smoking<br>Hypertension (BP ≥ 140/90 mmHg or on antihypertensive medication)<br>Low HDL-C (< 40 mg/dL) **<br>Family history of premature CHD (CHD in male first degree relative < 55 years; CHD in female first degree relative < 65 years)<br>Age (men ≥ 45 years, women ≥ 55 years)		

*In NCEP-ATP III, diabetes is regarded as a CHD risk equivalent. **HDL-C ≥ 60mg/dL counts as a "negative" risk factor: its presence removes one risk factor from the total count. LDL-C, low-density lipoprotein cholesterol; NCEP-ATP III, National Cholesterol. Education Program Adult Treatment panel Ⅲ; CHD, coronary heart disease; BP, blood pressure; HDL-C, high-density lipoprotein cholesterol

Supplementary Table S2. Changes in cholesterol profiles after 12 and 24 weeks of treatment with Rovazet® according to prior use of anti-dyslipidemic medication versus no prior use (treatment-naïve).

Lipid Profile		Efficacy Set(n = 5527)						
		Baseline	Week 12	% Change	p	Week 24	% Change	p
LDL-C, mg/dL					<0.0001			<0.0001
Prior-medication use	*n*	3503	3025	3017	<0.0001	3141	3138	<0.0001
	Mean(SD)	101.52 ± 46.29	76.76 ± 35.65	−15.45 ± 108.12		71.97 ± 32.04	−19.74 ± 65.34	
	Median	90.00	68.00	−20.00		66.00	−24.45	
	Min, Max	2.00, 501.00	3.70, 518.00	−92.76, 5,330.00		3.70, 350.90	−94.06, 2850.00	
Prior-medication naive	n	2012	1845	1844	<0.0001	1800	1798	<0.0001
	Mean(SD)	145.24 ± 45.67	88.32 ± 41.06	−36.63 ± 31.03		78.98 ± 35.12	−40.62 ± 32.06	
	Median	149.00	79.00	−38.91		72.00	−47.06	
	Min, Max	10.00, 375.00	3.00, 276.00	−98.01, 312.50		4.00, 249.00	−98.06, 342.31	
TC, mg/dL					<0.0001			<0.0001
Prior-medication use	*n*	3504	3024	3021	<0.0001	3139	3138	<0.0001
	Mean(SD)	177.38 ± 55.06	150.13 ± 43.57	−12.53 ± 20.92		145.32 ± 38.47	−14.42 ± 22.74	
	Median	166.00	140.00	−12.43		139.00	−15.13	
	Min, Max	64.00, 588.00	53.00, 391.00	−72.05, 121.43		55.00, 448.00	−76.59, 181.76	
Prior-medication naive	*n*	2001	1833	1833	<0.0001	1788	1788	<0.0001
	Mean(SD)	228.61 ±52.50	166.19 ± 47.58	−26.23 ± 20.92		155.52 ± 40.44	−29.17 ± 20.96	
	Median	232.00	159.00	−27.91		149.00	−33.18	
	Min, Max	72.00, 465.00	75.00, 410.00	−75.38, 93.02		65.00, 360.00	−70.36, 92.68	
TG, mg/dL					<0.0001			<0.0001
Prior-medication use	*n*	3507	3029	3024	<0.0001	3142	3140	<0.0001
	Mean(SD)	170.72 ±109.99	153.56 ± 109.30)	−0.17 ± 56.58		143.70 ± 84.73	−3.49 ± 56.49	
	Median	145.00	129.00	−10.54		125.00	−13.71	
	Min, Max	25.00, 1454.00	22.00, 2325.00	−91.54, 797.44		25.00, 1355.00	−90.08, 1361.11	
Prior-medication naive	*n*	2015	1847	1847	<0.0001	1802	1802	<0.0001
	Mean(SD)	187.52 ± 110.08	150.08 ± 83.00	−10.62 ± 46.78		141.30 ± 78.99	−13.75 ± 48.03	
	Median	162.00	133.00	−16.67		126.00	−22.73	

Lipid Profile		Efficacy Set(n = 5527)						
		**Baseline**	**Week 12**	**% Change**	***p***	**Week 24**	**% Change**	***p***
	Min, Max	26.00, 1046.00	29.00, 868.00	−92.08, 790.91		6.00, 871.00	−95.62, 578.57	
HDL-C, mg/dL					0.7034			0.5992
Prior-medication use	*n*	3502	3027	3020	<0.0001	3142	3139	<0.0001
	Mean(SD)	50.68 ± 15.86	51.31 ± 16.62	4.32 ± 28.92		52.21 ± 16.61	6.36 ± 37.79	
	Median	48.00	49.00	1.77		50.00	2.08	
	Min, Max	7.00, 280.00	5.00, 507.00	−92.06, 856.60		10.00, 500.00	−86.79, 1512.90	
Prior-medication naive	*n*	2012	1846	1846	<0.0001	1802	1799	<0.0001
	Mean(SD)	51.03 ± 15.10	51.86 ± 13.68	4.79 ± 31.51		52.27 ± 14.77	10.52 ± 66.19	
	Median	49.00	50.00	2.08		51.00	2.50	
	Min, Max	1.00, 173.00	4.00, 184.00	−94.94, 378.38		8.00, 270.00	−75.76, 1700.00	

Note. SD=Standard Deviation, Min=minimum, Max=maximum. 1) % Change=(Week 12 or 24-Baseline)/Baseline*100. 2) paired t-test or Wilcoxon's signed rank test(within group), ANOVA or Kruskal-Wallis test(between group)
